# Identification of protein interactions of grapevine fanleaf virus RNA-dependent RNA polymerase during infection of *Nicotiana benthamiana* by affinity purification and tandem mass spectrometry

**DOI:** 10.1099/jgv.0.001607

**Published:** 2021-05-27

**Authors:** Larissa J. Osterbaan, Victoria Hoyle, Michelle Curtis, Stacy DeBlasio, Keith D. Rivera, Michelle Heck, Marc Fuchs

**Affiliations:** ^1^​ Cornell University, Plant Pathology and Plant Microbe-Biology Section, School of Integrative Plant Science, Cornell AgriTech at the New York State Agricultural Experiment Station, Geneva, NY 14456, USA; ^2^​ Cornell University, Plant Pathology and Plant-Microbe Biology Section, School of Integrative Plant Science, Ithaca, NY 14853, USA; ^3^​ Emerging Pests and Pathogens Research Unit, USDA Agricultural Research Service, Robert W. Holley Center for Agriculture and Health, Ithaca, NY 14853, USA; ^4^​ Cold Spring Harbor Laboratory, Cold Spring Harbor, NY 11724, USA; ^†^​ Present address: Department of Biology, Utica College, Utica, NY 13502, USA; ^‡^​ Present address: The Broad Institute of Massachusetts Institute of Technology and Harvard, Cambridge, MA 02142, USA

**Keywords:** affinity purification, grapevine fanleaf virus, *Nepovirus*, *Nicotiana benthamiana*, RNA-dependent RNA polymerase, *Secoviridae*

## Abstract

The RNA-dependent RNA polymerase (1E^Pol^) is involved in replication of grapevine fanleaf virus (GFLV, *Nepovirus*, *Secoviridae*) and causes vein clearing symptoms in *Nicotiana benthamiana*. Information on protein 1E^Pol^ interaction with other viral and host proteins is scarce. To study protein 1E^Pol^ biology, three GFLV infectious clones, i.e. GHu (a symptomatic wild-type strain), GHu-1E_K802G_ (an asymptomatic GHu mutant) and F13 (an asymptomatic wild-type strain), were engineered with protein 1E^Pol^ fused to a V5 epitope tag at the C-terminus. Following *Agrobacterium tumefaciens *-mediated delivery of GFLV clones in *N. benthamiana* and protein extraction at seven dpi, when optimal 1E^Pol^:V5 accumulation was detected, two viral and six plant putative interaction partners of V5-tagged protein 1E^Pol^ were identified for the three GFLV clones by affinity purification and tandem mass spectrometry. This study provides insights into the protein interactome of 1E^Pol^ during GFLV systemic infection in *N. benthamiana* and lays the foundation for validation work.

Grapevine fanleaf virus (GFLV) is a member of the genus *Nepovirus* in the family *Secoviridae* [1–4]. Its two positive-sense single-stranded genomic RNAs carry a genome-linked viral protein (VPg) at their 5′ end and are polyadenylated at their 3′ end [1, 3]. Expression of the two genomic RNAs is by monocistronic translation and proteolytic processing [1, 3]. GFLV RNA1 encodes five proteins for genome replication and polyprotein maturation, including protein 1A (46 kDa) of unknown function [3], a putative ATP-dependent helicase with membrane- and nucleoside triphosphate-binding motifs (protein 1B^Hel^, 88 kDa) [3, 5], a genomic-linked protein (protein 1C^VPg^, 3 kDa) [3, 5], a cysteine protease (protein 1D^Pro^, 24 kDa) [3, 6] and the RNA-dependent RNA polymerase (protein 1E^Pol^, 92 kDa) [3, 5, 7]. RNA2 encodes three proteins for RNA2 replication (protein 2A^HP^), movement (protein 2B^MP^) and encapsidation (protein 2C^CP^) [1, 3]. Systemic GFLV infection *in planta* requires both RNA1 and RNA2 [8].

Replication of GFLV occurs on endoplasmic reticulum-derived vesicles in infected plant cells and requires *de novo* lipid synthesis [3]. GFLV RNA1-encoded protein 1C^VPg^ and RNA2-encoded protein 2A^HP^ localize to the perinuclear replication compartments that contain double-stranded RNA molecules [3]. Little is known about the involvement of other viral proteins in replication although GFLV RNA1-encoded proteins 1E^Pol^ and 1B^Hel^ are suspected to be essential [3]. In addition, no information is available on the interaction of protein 1E^Pol^ with other GFLV proteins and host proteins for replication.

Similarly, the molecular mechanisms underpinning GFLV symptom development remain largely unknown although recent advances have been made in model herbaceous hosts. For example, GFLV strain F13 produces a hypersensitive response triggered by the RNA2-encoded protein 2A^HP^ on inoculated leaves of *Nicotiana occidentalis* [9]. In contrast, GFLV-F13 produces an asymptomatic infection in *N. benthamiana,* while GFLV strain GHu produces distinct vein clearing symptoms on apical leaves of this plant species [7, 10]. A symptom determinant for vein clearing of GFLV-GHu was recently mapped to residue 802 of the RNA1-encoded protein 1E^Pol^. This residue, which is a lysine in GFLV-GHu, is necessary but not sufficient for vein clearing development [10].

While much work has been done to describe molecular events of GFLV infection [3, 11], information about the molecular context of protein 1E^Pol^ during infection, in particular its protein interactants, is lacking. Here, we built on our previous work and developed a method to isolate protein 1E^Pol^ from protein extracts of *N. benthamiana* infected with GFLV and initiated a proof-of-concept study of the 1E^Pol^ protein interactome via affinity purification coupled to tandem mass spectrometry. To this end, tagging GFLV protein 1E^Pol^ was essential because, in our hands, efforts to generate an antibody that specifically detected 1E^Pol^ were unsuccessful. Indeed, an antibody raised against a synthetic peptide (HVPSKTSFMKVPDELC) designed in a conserved N-terminus sequence failed to unambiguously detect an immunoreactive product of the expected size in total soluble protein extracts of GFLV-infected *N. benthamiana* via SDS-PAGE and western blot detection, although the same approach was successful in a previous study [7]. Thus, we decided to tag protein 1E^Pol^ as an alternative to producing an antibody against 1E^Pol^.

The C-terminus of GFLV protein 1E^Pol^ was tagged by inserting the sequence of one of five common epitope tags, i.e. V5 [12], FLAG [13], 3XFLAG [14], HA [15] or myc [16] (Table S1, available with the online version of this article), in GFLV RNA1 cDNA constructs using the Q5 Site-Directed Mutagenesis Kit (New England Biolabs). Plasmids pCLEAN-F131-35S, pCLEAN-GHu1-35S and pCLEAN-GHu-1E_K802G_-35S [10] served as PCR and cloning templates for tagging experiments using specific primers (Table S2). The GFLV RNA1 cDNA constructs are cloned within a cauliflower mosaic virus 35S expression cassette for expression *in planta* [10, 12]. Following *Agrobacterium tumefaciens *-mediated delivery of recombinant pCLEAN GFLV RNA1 constructs in the presence of pCLEAN-GHu-2–35S by syringe infiltration of *N. benthamiana* leaves [10, 17], only V5-tagged GFLV strains GHu and GHu-1E_K802G_, an asymptomatic mutant of GHu for which the lysine in position 802 of protein 1E^Pol^ was substituted by a glycine [10], established systemic infection. None of the other GFLV-GHu recombinant clones were infectious *in planta* (Table S3). Typical vein clearing symptoms were observed in apical leaves for GFLV-GHu but no symptoms were apparent for GHu-1E_K802G_ (Fig. 1a). These phenotypes were consistent with those of untagged viruses [7, 10, 17]. In addition, GFLV-GHu and its mutant were detected in uninoculated, apical *N. benthamiana* leaves by DAS-ELISA using GFLV specific antibodies (Bioreba 120642) and their 1E^Pol^:V5 protein (~93 kDa) accumulated in protein extracts from apical leaves, as shown by SDS-PAGE and western blot detection with an anti-V5 antibody (Invitrogen PA1-993) (Fig. 1b, red arrowhead). In addition, an anti-V5 immunoreactive protein band of higher molecular mass (~117 kDa) was apparent in western blot for GFLV-GHu and GHu-1E_K802G_ (Fig. 1b, green arrowhead). This protein was detected in the affinity purifications using cross-absorbed and non-cross-absorbed antibodies. It could correspond to the 1D^Pro^E^Pol^ (117 kDa) or 1C^VPg^1D^Pro^1E^Pol^ (120 kDa) precursors (Fig. 1b, c).

**Fig. 1.**
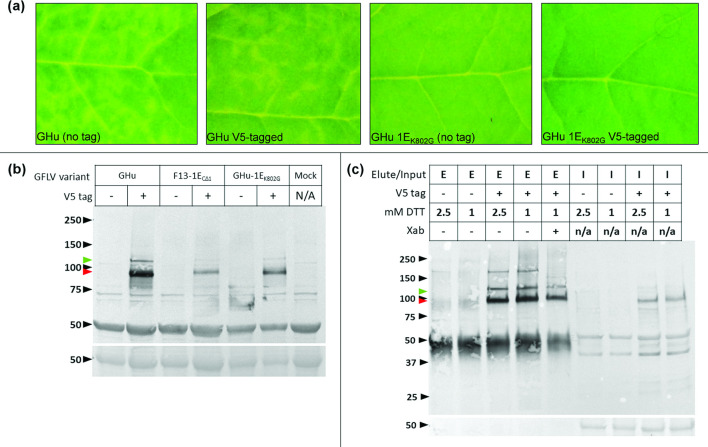
(**a**) Symptom development in *Nicotiana benthamiana* plants infected with GFLV strain GHu or GHu-1E_K802G_, an asymptomatic mutant of GFLV-GHu in which lysine 802 of protein 1E^Pol^ was mutated to glycine, and their corresponding version for which protein 1E was tagged with a V5 epitope. Insets show close-ups of vein clearing symptoms (first and second panels) and asymptomatic leaves (third and fourth panels) at six dpi. (**b**) Detection of protein 1E^Pol^:V5 accumulation in *N. benthamiana* leaf tissue systemically infected with wild-type (-) or V5-tagged 1E^Pol^ (+) GFLV strains GHu and F13-1E_CΔ1_, and mutant GHu-1E_K802G_ by western blot with an anti-V5 antibody. TSP from a mock-inoculated *N. benthamiana* were used as a control. (**c**) Analysis of affinity purified 1E^Pol^ complexes in TSP from *N. benthamiana* leaves systemically infected with GFLV-GHu or GFLV-GHu-1E:V5 by western blot. TSP were extracted in lysis buffer 4 amended with 1X Halt protease inhibitor cocktail (Thermo Fisher Scientific) and further diluted 1 : 5 in DTT-free lysis buffer. Inputs were affinity purified with V5 polyclonal antibody (Invitrogen PA1-993) conjugated to Protein A Dynabeads (Invitrogen). The top row of lane labels indicates (**e**) elution products or (**i**) input fractions of the affinity purification. The second row indicates V5-tagged (+) or non-tagged (-) GFLV-GHu. The third row indicates the concentration of DTT included in the lysis buffer. The fourth row indicates whether the V5 polyclonal antibody used for affinity purification was untreated (-) or cross-absorbed against TSP from healthy *N. benthamiana* tissue (+). Proteins were probed with a polyclonal anti-V5 antibody (Invitrogen PA1-993, non-cross-absorbed) and a goat anti-rabbit alkaline phosphatase-conjugated secondary antibody (Invitrogen T2191) and developed with 1-Step NBT/BCIP solution. The predicted molecular weight of (**i**) V5-tagged 1E^Pol^ (93 kDa) is indicated with a red arrowhead, and (ii) a putative V5-tagged 1D^Pro^E^Pol^ (117 kDa) or 1C^VPg^1D^Pro^E^Pol^ (120 kDa) precursor is indicated with a green arrowhead. Bottom panels show Ponceau staining of the RuBisCO large subunit. Molecular weight standards (kDa) are shown by black arrowheads.

For GFLV strain F13, a similar epitope tagging approach abolished infectivity *in planta*. Only mutants GFLV-F13-1E_CΔ1_:V5 and GFLV-F13-1E_CΔ4_:V5, in which a single or four residues were truncated at the C-terminus of protein 1E^Pol^ prior to the addition of the V5 epitope tag, established a systemic infection in *N. benthamiana*, as shown by DAS-ELISA using specific antibodies (Table S3). Infection of GFLV-F13-1E_CΔ1_:V5 and GFLV-F13-1E_CΔ4_:V5 was asymptomatic, consistent with the phenotype of the untagged virus [7, 10, 17]. Less 1E^Pol^:V5 accumulated in *N. benthamiana* infected with GFLV-F13-1E_CΔ1_:V5 compared to GFLV-GHu:V5 and GFLV-GHu1E_K802G:_V5 following infection (Fig. 1b). More work is needed to explain this differential accumulation of protein 1E^Pol^:V5 between GFLV strains GHu and F13. Of the two GFLV-F13 tagged mutants only F13-1E_CΔ1_:V5 reliably showed an anti-V5 immunoreactive 1E^Pol^:V5 signal in protein extracts from apical *N. benthamiana* leaves tested by western blot detection (Table S3). Thus, this mutant was further used in a comparative proteomics analysis of tagged and untagged virus isolates.

GFLV recombinants carrying protein 1E^Pol^ with the epitope tags FLAG, 3XFLAG, HA or myc repetitively failed to establish systemic infection in *N. benthamiana* (Table S3). Interestingly, of the five epitope tags tested, V5 has the lowest proportion of acidic residues. The V5 epitope (1.4 kDa) has 7 % acidic residues in contrast to the FLAG, 3XFLAG, HA and myc tags, which have 63, 50, 40 and 22 % acidic residues, respectively (Table S1). A low proportion of acidic residues is consistent with the residue composition of the C-terminal 53 amino acids of protein 1E^Pol^ of GFLV strains GHu (1.9 % acidic residues) and F13 (3.7 % acidic residues), suggesting the functionality of protein 1E^Pol^ may depend on the charge of its C-terminus. It is also possible that epitope tags, except V5, might have affected the stability of protein 1E^Pol^ or its capacity to be properly translated. More work is needed to address these issues.

A replicated time course experiment in *N. benthamiana* via mechanical inoculation with GFLV-GHu-1E^Pol^:V5 showed optimal accumulation of immunoreactive 1E^Pol^:V5 in TSP of apical leaves at 6–9 dpi by SDS-PAGE and western blotting with an anti-V5 antibody. This optimal period corresponds with noticeable GFLV-GHu vein clearing symptoms at 4–6 dpi (Fig. 1a) and symptoms fading at 9–10 dpi.

Of the four lysis buffers [18–21] (Table S4) tested for the extraction of 1E^Pol^:V5 at seven dpi, a reducing agent such as DTT was necessary to confidently detect an immunoreactive peptide (Fig. 2a). It may be that a reducing environment is necessary to dissociate protein 1E^Pol^ from membranes, nucleic acids or other macromolecules. In support of this hypothesis, GFLV 1E^Pol^ likely associates with endoplasmic reticulum-derived membranes for replication [3, 11, 22], although it might not bind to membranes by itself when expressed ectopically in *N. benthamiana*, suggesting a probable dependence on another viral protein, likely protein 1B^Hel^, to anchor to the replication complex, as previously discussed [3]. Similarly, the polymerase domain-containing precursor protein of tomato ringspot virus, another nepovirus, associates with endoplasmic reticulum-derived membranes [23].

**Fig. 2.**
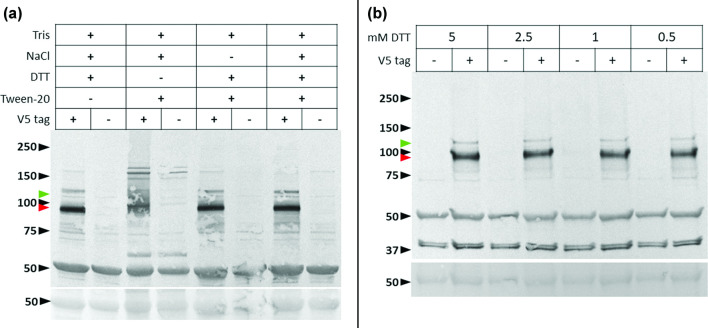
Optimization of lysis buffer and extraction conditions of V5-tagged grapevine fanleaf virus (GFLV) protein 1E^Pol^ from systemically infected *Nicotiana benthamiana* tissue. (**a**) TSP extracted with lysis buffer one and lysis buffer one without (-) NaCl, DTT, or Tween-20. Single elimination of components from lysis buffer identified DTT as necessary for efficient detection of GFLV 1E^Pol^ from *N. benthamiana* tissue. (**b**) TSP extracted using lysis buffer 4 amended with 5.0 mM or 2.5 mM DTT, followed by a 1 : 5 post-extraction dilution with DTT-containing lysis buffer 4 (final DTT concentration of 5 and 2.5 mM, respectively) or DTT-free lysis buffer 4 (final DTT concentration of 1 and 0.5 mM, respectively). Top images show western blots of total soluble proteins (TSP) extracted from cryogenically milled *N. benthamiana* leaves systemically infected with either GFLV-GHu containing V5-tagged 1E^Pol^ (+) or wild-type GFLV-GHu 1E^Pol^ (non-tagged, -). Bottom images show Ponceau staining of the RuBisCO large subunit. Proteins were probed as described in Fig. 1. Molecular standards (in kDa) are shown by black arrowheads. The predicted molecular weight of V5-tagged 1E^Pol^ (93 kDa) is indicated with a red arrowhead, and that of a putative V5-tagged 1D^Pro^E^Pol^ (117 kDa) or 1C^VPg^1D^Pro^E^Pol^ (120 kDa) precursor is indicated with a green arrowhead.

Affinity purifications of the 1E^Pol^:V5 protein complexes were performed on TSP extracted from *N. benthamiana* systemically infected with GFLV-GHu-1E:V5, GFLV-F13-1E_CΔ1_:V5 or GFLV-GHu-1E_K802G_:V5 collected at seven dpi. Untagged GFLV strains GHu and F13 were used as negative controls. Three biological replicates were performed for each virus treatment. Affinity purification conditions were as previously described [18] with the following modifications. The anti-V5 tag polyclonal antibody was bound to Dynabeads Protein A (Life Technologies, Invitrogen) at a concentration of 10 µg antibody per 1 mg of beads and 5 mg of beads in 5 ml of diluted plant cell lysate were used. For protein extraction, lysis buffer 4 (Table S4), a HEPES-based lysis buffer, was supplemented with 2.5 mM DTT, the lowest concentration of DTT required for sufficient extraction of 1E^Pol^:V5, and lysates were further diluted 1 : 5 in lysis buffer without DTT (Fig. 2) to minimize loss of 1E^Pol^-plant protein interactions and inhibition of bait capture by the V5 antibody, respectively. Washes of beads were performed with DTT-free lysis buffer and the full bead volume was subjected to on-bead trypsin digestion, as described [18]. Additionally, to enrich for 1E^Pol^:V5 and reduce background binding during affinity purifications, the anti-V5 antibody was cross-absorbed by sodium sulphate precipitation [24] with proteins from a blend of *N. benthamiana* tissue infected with untagged GFLV-GHu, F13 and GHu-1E_K802G_ in equal proportions. The antibody fraction was recovered by overnight dialysis, as previously described [18]. Cross-absorption of the anti-V5 antibody resulted in the loss of some anti-V5 immunoreactive protein bands of higher molecular mass in western blot detection (Fig. 1c).

Sample cleaned for mass spectrometry was performed using OMIX C18 100 µl tips (Agilent, A57003100). After trypsin digestion, peptides were eluted from beads in 100 µl 0.1 % formic acid. Protein complexes were analysed on an Orbitrap Fusion Lumos mass spectrometer (Thermo Scientific) equipped with a nano-ion spray source coupled to an EASY-nLC 1200 system (Thermo Scientific). The liquid chromatography system was configured with a self-pack PicoFrit 75 -µm analytical column with an 8 -µm emitter (New Objective, Woburn, MA) packed to 25 cm with ReproSil-Pur C18-AQ, 1.9 µm material (Dr. Maish HPLC, GmbH).

Thermo RAW files were converted to Mascot generic files (.mgf) using ProteoWizard [25]. The protein search database was generated from amino acid sequences corresponding to all coding gene sequences from version 1.01 of the *N. benthamiana* genome assembly downloaded from the Sol Genomics Network [26], amino acid sequences from nepoviruses including mutant and natural strains of GFLV, plus common mammalian affinity purification contaminant proteins downloaded from NCBI. The Mascot v. 2.5.1 software used to search the mgf files identified an average of 722 protein groups per replicate. Search parameters included one fixed modification (cysteine: carbamidomethyl), two variable modifications (deamidation of asparagine and glutamine and/or methionine oxidation), trypsin enzyme specificity, one missed cleavage, a peptide mass tolerance of ±20 ppm, fragment mass tolerances of ±0.5 Da, and ion charge=2+, 3+ or 4+.

Data were imported into Scaffold Q+4.80.4 (Proteome Software Inc., Portland, OR) for spectrum counting analysis. Peptide and protein false discovery rates were set at <1.0 % using the Peptide Prophet algorithm [27] with delta-mass correction. Protein identifications were accepted if they contained at least two identified peptides. Protein probabilities were assigned by the Protein Prophet algorithm [27]. Proteins that contained similar peptides and could not be differentiated based on tandem mass spectrometry analysis alone were grouped to satisfy the principles of parsimony.

Spectral counts for proteins in the tagged (*n*=9 biological replicates, each from independent plants) and untagged (*n*=5 biological replicates, each from independent plants) samples were compared using a Student’s *t*-test to identify proteins that may bind to protein 1E^Pol^, but not in a GFLV strain specific manner, because no host proteins were found to be enriched when each virus strain was tested individually. Log_2_ fold-change enrichment (tagged/untagged) was calculated to determine enrichment in the experiments with the tagged 1E^Pol^.

Three RNA1-encoded GFLV proteins were significantly enriched in the affinity purification experiments: 1E^Pol^, 1B^Hel^ and 1D^Pro^ (Table 1). A single peptide spectral matching to protein 1E^Pol^ was detected at low levels in three of the five untagged replicates (Table 1), consistent with minimal levels of non-specific binding of 1E^Pol^ to the beads or the anti-V5 antibody used in the affinity purifications. In contrast, 1E^Pol^ was abundantly detected in the tagged replicates, as expected, and proteins 1B^Hel^ and 1D^Pro^ were only detected in experiments with tagged protein 1E^Pol^ (Table 1). A total of six plant proteins were identified to co-purify with GFLV protein 1E^Pol^ at low levels (Table 1). Among the six plant proteins, three proteins were specifically found in the tagged 1E^Pol^ samples: plastid transcriptionally active 14 Set domain protein (pTAC14), splicing factor 3B subunit one protein and dynamin-related protein 5A. The other three plant proteins, WD40 domain-containing protein, translation initiation factor IF-2 protein and P-type ATPase (PMA1), were detected in both the tagged and untagged samples but were found to be enriched (1.2 to 2.8 Log_2_-fold) in the tagged samples with a *P*-value <0.05 (Table 1).

**Table 1.** Potential protein interaction partners of grapevine fanleaf virus RNA1-encoded protein 1E^Pol^ identified using affinity purification coupled to tandem mass spectrometry

Accession number*	Protein Annotation†	Fold-change enrichment/Spectral counts‡	*P*-value§	Peptide Sequence||	Peptide identification Probability¶	Mascot ion Score#	Mascot identity threshold
AFM91094	GFLV_1B^Hel^	∞ (26/0)	0.01723	K/NLLGEHILAEEEK/L	100	74.1	34.1
AFM91094	GFLV_1D^Pro^	∞ (48/0)	0.02712	R/GVTYSSVIPSYSSSYVR/-	100	72.6	37.1
NP_619689	GFLV_1E^Pol^	6.364 (716/4)	0.04665	K/LLDNVNTALVELYLHGDR/T	100	126.1	30.9
Niben101Scf03607g00009	Plastid transcriptionally active 14 SET domain protein	∞ (15/0)	0.00576	K/VIQALDIYQDR/I	100	49.3	35.7
Niben101Scf06128g00004	Splicing factor 3B subunit 1	∞ (19/0)	0.03184	R/LGETFNETAIPLR/Y	100	55.9	34.2
Niben101Scf01006g03016	Dynamin-related protein 5A	∞ (20/0)	0.04766	R/VEVNGAAVESLER/M	100	60.7	37.5
Niben101Scf01814g05011	WD40 domain-containing protein	2.8 (38/3)	0.02337	K/LDLSEILYQITSR/F	100	61	31.6
Niben101Scf04225g02007	Translation initiation factor IF-2	1.7 (30/5)	0.03922	K/VAASEAGGITQGIGAYK/V	100	72.8	34.5
Niben101Scf00593g01002	P-type ATPase (PMA1)	1.2 (59/14)	0.04770	K/LFSEATNFNELNQLAEEAK/R	100	93.5	37

*Sequence accession number from NCBI (for viral proteins) and Sol Genomics databases (for plant proteins).

†Protein annotations derived from NCBI for viral proteins and manual curation using blast for plant proteins.

‡Fold-change enrichment for proteins computed as Log_2_ (spectral counts in tagged samples/spectral counts in untagged samples).

§*P*-values computed using a Student’s *t*-test.

||Sequence of the top-scoring peptide used to identify the protein.

¶Estimate of Scaffold peptide identification probability based on the quality of the MS/MS spectrum to peptide sequence match.

#The ion score for an MS/MS match in Mascot is based on the calculated probability, P, that the observed match between the experimental data and the database sequence is a random event. The reported score is −10Log(P). Ion scores that are higher than the Mascot identity threshold indicate high quality matches.

The Mascot peptide identity threshold is the ion score with expected significance threshold to be 0.05.

Three of the plant proteins identified in complex with 1E^Pol^ have been shown to play a role in plant or animal virus infection. For example, protein pTAC14 is localized in the chloroplast and regulates plastid gene expression [28]; several virus proteins have been shown to be involved in a plasma membrane and chloroplast signalling pathway to suppress salicylic acid-dependent plant defenses [29]. In rice, the splicing factor 3B subunit one protein regulates the expression of genes involved in cell death and resistance responses [30]. Splicing factor 3B subunit one has also been characterized to interact with animal viruses and plays a critical role in the replication of human immunodeficiency virus [31]. Dynamin-related protein 5A is critical for plant infection by soybean mosaic virus (SMV, genus *Potyvirus*, family *Potyviridae*) [32]. Dynamin-related protein 5A was identified in purifications of SMV virions using proteomics and knock-down of dynamin-related protein 5A in plants inhibited SMV infection in soybean [32]. Most of the plant proteins reported in this study were also described in the interactome of purified potato leafroll virus (genus *Polevirus*, family *Luteovidae*) virions during infection of *N. benthamiana* [18]. Additionally, recent evidence supports a role for PMA1 in the induction of an immune response manifested by cell death in *N. benthamiana*, as shown by silencing and overexpression assays [33].

Our proof-of-concept study based on affinity purifications of V5-tagged 1E^Pol^ coupled with protein identification by tandem mass spectrometry provided a snapshot of the putative protein interaction network of protein 1E^Pol^ during systemic GFLV infection of *N. benthamiana*. Methods to optimize the extraction of protein 1E^Pol^ from plant tissue may increase the depth of proteomic coverage and lead to a more thorough characterization of the plant-virus interactome. GFLV protein 1D^Pro^, the viral protease which processes *in cis* and *in trans* the two viral polyproteins into individual mature peptides [1, 3, 5, 6, 11], was enriched in affinity purifications with V5-tagged 1E^Pol^, suggesting that 1D^Pro^ complexes with 1E^Pol^ during virus infection. The 1B^Hel^ helicase protein [1, 3, 5, 11] was also enriched as a protein interacting in complex with 1E^Pol^ during virus infection. It is also possible that protein 1D^Pro^ peptides are enriched in affinity purifications with 1E^Pol^ due to the presence of putative 1D^Pro^1E^Pol^ or 1C^VPg^1D^Pro^1E^Pol^ precursors of polyprotein processing that were suggested by western blot analyses (Figs 1 and 2). More research is needed to test whether interactions between GFLV proteins 1E^Pol^, 1D^Pro^ and 1B^Hel^ are direct or indirect, to understand the stoichiometry of binding and the significance of these interactions during virus infection, in particular in replication.

Orthologous of most plant host proteins enriched in our data set were previously documented to be involved in virus-host interactions with other plant or animal viruses [18, 28–32]. These host protein candidates will need to be validated in follow-up genetic approaches to verify whether they have a role in GFLV infection in *N. benthamiana*.

## Supplementary Data

Supplementary material 1
