# Supplementary material for: Identification of protein interactions of grapevine fanleaf virus RNA-dependent RNA polymerase during infection of Nicotiana benthamiana by affinity purification and tandem mass spectrometry
Source: J Gen Virol. 2021 May 27;102(5):001607. doi: 10.1099/jgv.0.001607 (PMC8295916; doi:10.1099/jgv.0.001607)
Supplement: Supplementary material 1 [file jgv-102-1607-s001.pdf]

Supplementary Table 1. Five epitope tags used in this study to label the C-terminus of protein $1E^{Pol}$ of grapevine fanleaf virus, their amino acid sequence and composition in acidic residues.

Epitope tag	Source	Amino acid sequence	Acidic residue	Reference
V5	Simian virus V5	GKPIPNPLLGLDST	1 of 14 (7%)	[26]
FLAG	Synthetic	DYKDDDDK	5 of 8 (63%)	[27]
3XFLAG	Synthetic	DYKDHDGDYKDHDIDYKDDDDK	11 of 22 (50%)	[28]
HA	Human influenza virus	YPYDVPDYA	4 of 10 (40%)	[29]
myc	Human	EQKLISEEDL	2 of 9 (22%)	[30]

Supplementary Table 2. Primers used to insert epitope tag sequences into the coding region of protein $1E^{Pol}$ in grapevine fanleaf virus RNA1 cDNA constructs of strains GHU and F13.

Construct	Template	Forward primer	Forward primer sequence (5'-3')[a]	Reverse primer	Reverse primer sequence (5'-3')[a]
pCLEAN-GHu-1E:3XFLAG	pCLEAN-GHu-1	GHu-1E-3XFLAG-F	catgacatcgattacaaggatgacgatgacaagTAATTCTTCCAACCCTTGGTAC	GHu-1E-3XFLAG-R	atctttataatcaccgtcatggtctttgtagtcCTTCCTCGGGCATGAGTG
pCLEAN-GHu-1E:FLAG	pCLEAN-GHu-1	GHu-1E-FLAG-F	gatgatgataaaTAATTCTTCCAACCCTTGGTAC	GHu-1E-FLAG-R	atctttataatcCTTCCTCGGGCATGAGTG
pCLEAN-GHu-1E:HA	pCLEAN-GHu-1	GHu-1E-HA-F	gccggattatgcgTAATTCTTCCAACCCTTGGTAC	GHu-1E-HA-R	acatcatacggataCTTCCTCGGGCATGAGTG
pCLEAN-GHu-1E:myc	pCLEAN-GHu-1	GHu-1E-myc-F	agcgaagaagatctgTAATTCTTCCAACCCTTGGTAC	GHu-1E-myc-R	aatcagtttctgttcCTTCCTCGGGCATGAGTG
pCLEAN-GHu-1E:V5	pCLEAN-GHu-1	GHu-1E-V5-F	ctgctgggcctggatagcaccTAATTCTTCCAACCCTTGGTAC	GHu-1E-V5-R	cgggttcggaatcggtttgccCTTCCTCGGGCATGAGTG
pCLEAN-GHu-$1E_{K802G}$:V5	pCLEAN-GHu-1E-K802G	GHu-1E-V5-F	ctgctgggcctggatagcaccTAATTCTTCCAACCCTTGGTAC	GHu-1E-V5-R	cgggttcggaatcggtttgccCTTCCTCGGGCATGAGTG
pCLEAN-F13-1E:3XFLAG	pCLEAN-F13-1	F13-1E-3XFLAG-F	catgacatcgattacaaggatgacgatgacaagTAAGCCTTCCAATTCTTG	F13-1E-3XFLAG-R	atctttataatcaccgtcatggtctttgtagtcTTTCCTAAGGCATGTATG
pCLEAN-F13-1E:FLAG	pCLEAN-F13-1	F13-1E-FLAG-F	gatgatgataaaTAAGCCTTCCAATTCTTG	F13-1E-FLAG-R	atctttataatcTTTCCTAAGGCATGTATG
pCLEAN-F13-1E:HA	pCLEAN-F13-1	F13-1E-HA-F	gccggattatgcgTAAGCCTTCCAATTCTTG	F13-1E-HA-R	acatcatacggataTTTCCTAAGGCATGTATG
pCLEAN-F13-1E:myc	pCLEAN-F13-1	F13-1E-myc-F	agcgaagaagatctgTAAGCCTTCCAATTCTTG	F13-1E-myc-R	aatcagtttctgttcTTTCCTAAGGCATGTATG
pCLEAN-F13-1E:V5	pCLEAN-F13-1	F13-1E-V5-F	ctgctgggcctggatagcaccTAAGCCTTCCAATTCTTG	F13-1E-V5-R	cgggttcggaatcggtttgccTTTCCTAAGGCATGTATG
pCLEAN-F13-$1E_{\Delta C1}$:V5	pCLEAN-F13-1	F13-1E-V5-F	ctgctgggcctggatagcaccTAAGCCTTCCAATTCTTG	F13-1E-V5:C-1_Rev	cgggttcggaatcggtttgccCCTAAGGCATGTATGATTC

[a]Lower case type indicates mutagenic (insertion) sequences. Upper case type indicates residues which anneal to the original template.

Supplementary Table 3. List of wild-type and chimeric grapevine fanleaf virus (GFLV) strains used in this study and some of their biological properties.

GFLV strain	Chimeric 1E construct[a]	Infectivity in *Nicotiana benthamiana*[c] Inoculated leaves	Apical leaves	Detection of $1E^{Pol}$ by western blot[d]
GHu	1E:V5	+	+	+
	1E:FLAG	-	-	n/a
	1E:3XFLAG	-	-	n/a
	1E:HA	-	-	n/a
	1E:myc	-	-	n/a
	$1E_{K802G}$:V5[b]	+	+	+
F13	1E:V5	-	-	n/a
	$1E_{C\Delta1}$:V5	+	+	+
	$1E_{C\Delta2}$:V5	-	-	n/a
	$1E_{C\Delta3}$:V5	-	-	n/a
	$1E_{C\Delta4}$:V5	+	+	+/-

[a]GFLV protein $1E^{Pol}$ was tagged with the V5, FLAG, 3XFLAG, HA, or myc epitope.

[b]GFLV-GHU-$1E_{K802G}$:V5 is a mutant of GFLV-GHu for which the lysine in position 802 of protein 1E is substituted by a glycine.

[c]GFLV infection was determined in inoculated and uninoculated, apical leaves of *Nicotiana benthamiana* by DAS-ELISA using specific antibodies and by monitoring vein clearing symptom development in apical leaves. Presence (+) or absence (-) of GFLV was recorded.

[d]Western blot assays were performed with total soluble proteins isolated from infected and mock inoculated plants, and a commercially available anti-V5 tag antibody (Invitrogen PA1-993); n/a: not applicable; consistent detection of protein $1E^{Pol}$:V5 (+); and inconsistent detection of protein $1E^{Pol}$:V5 (+/-).

Supplementary Table 4. Composition of four lysis buffers used in this study to extract total soluble proteins from *Nicotiana benthamiana* plants infected with grapevine fanleaf virus (GFLV) for the detection of protein $1E^{Pol}$:V5 by western blot with a polyclonal anti-V5 antibody.

Lysis buffer	Composition[a]	Reference
1	50 mM Tris-HCl (pH 8.0), 100 mM NaCl, 10 mM DTT, 0.1% Tween-20	[31]
2	100 mM Tris-HCl (pH 8.1), 10 mM KCl, 5 mM $MgCl_2$, 400 mM sucrose, 10% glycerol, 1 mM β-mercaptoethanol	[32]
3	50 mM Tris-HCl (pH 7.6), 150 mM NaCl, 10% glycerol, 1 mM DTT, 0.1% octylphenoxy poly(ethyleneoxy)ethanol	[33]
4	50 mM HEPES-KOH (pH 7.4), 110 mM KOAc, 2 mM $MgCl_2$, 0.4% TritonX-100	[15]

[a]All buffers were amended with 1X Halt protease inhibitor cocktail (Thermo Fisher Scientific).

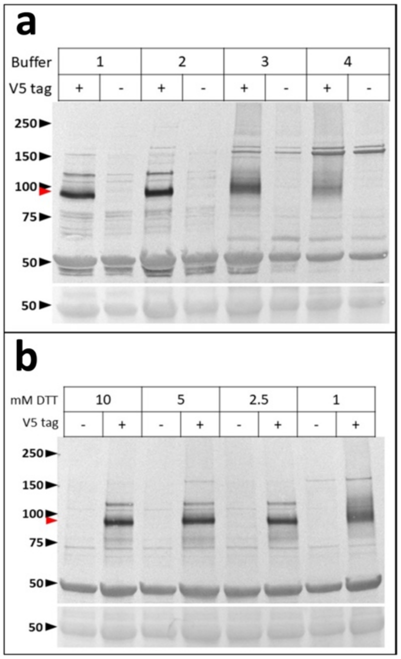

Supplementary Figure 1. Effect of buffer formulation on the extraction of V5-tagged grapevine fanleaf virus (GFLV) protein 1E^{Pol} from systemically infected *Nicotiana benthamiana* tissue for detection by western blot using a polyclonal anti-V5 antibody. Top images show western blots of total soluble proteins (TSP) extracted from cryogenically milled *N. benthamiana* leaves systemically infected with either GFLV-GHu containing V5-tagged 1E^{Pol} (+) or wild type GFLV-GHu 1E^{Pol} (non-tagged, -). Bottom images show Ponceau staining of the RuBisCO large subunit. Proteins were probed with a polyclonal anti-V5 antibody (Invitrogen PA1-993, non-cross-absorbed) and goat anti-rabbit AP-conjugated secondary antibody (Invitrogen T2191) and developed with 1-Step NBT/BCIP solution. Molecular standards (in kDa) are shown by black arrowheads. The predicted molecular weight of V5-tagged 1E^{Pol} (93 kDa) is indicated with a red arrowhead. **A.** TSP extracted using one of four lysis buffers 1-4 (Supplementary Table 1). All buffers were amended with 1X Halt protease inhibitor cocktail (Thermo Fisher Scientific). **B.** TSP extracted using buffer 4 amended with varying concentrations (10, 5, 2.5, or 1 mM) of DTT. Optimal detection of V5-tagged GFLV 1E^{Pol} was achieved with lysis buffer 4 amended with 2.5 mM DTT.
